# Discharging of Ramsdellite MnO_2_ Cathode in a Lithium-Ion Battery

**DOI:** 10.1021/acs.chemmater.4c01417

**Published:** 2024-08-30

**Authors:** Woongkyu Jee, Alexey A. Sokol, Cyril Xu, Bruno Camino, Xingfan Zhang, Scott M. Woodley

**Affiliations:** Department of Chemistry, University College London, 20 Gordon Street, London WC1H 0AJ, U.K.

## Abstract

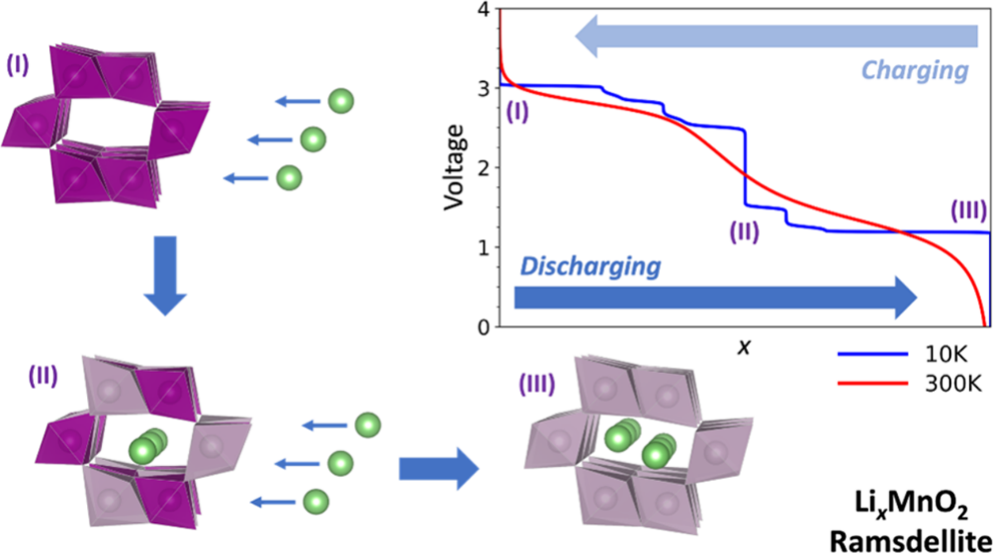

We report an application of our unbiased Monte Carlo approach to investigate thermodynamic and electrochemical properties of lithiated manganese oxide in the ramsdellite phase (R-MnO_2_) to uncover the mechanism of lithium intercalation and understand charging/discharging of R-MnO_2_ as a cathode material in lithium-ion batteries. The lithium intercalation reaction was computationally explored by modeling thermodynamically significant distributions of lithium and reduced manganese in the R-MnO_2_ framework for a realistic range of lithium molar fractions 0 < *x* < 1 in Li_*x*_MnO_2_. We employed interatomic potentials and analyzed the thermodynamics of the resultant grand canonical ensemble. We found ordered or semiordered phases at *x* = 0.5 and 1.0 in Li_*x*_MnO_2_, verified by configurational entropy changes and simulated X-ray diffraction patterns of partially lithiated R-MnO_2_. The radial distribution functions show the preference of lithium for homogeneous distributions across the one-dimensional 2 × 1 ramsdellite channels accompanied by alternating reduced/oxidized manganese ions. The occupation of the interstitial sites in the channels is correlated with the calculated voltage profile, showing a sharp voltage drop at *x* = 0.5, which is explained by the energy penalty of shifting lithium ions from stable tetrahedral to unstable octahedral sites. To facilitate this work, our in-house software, Knowledge Led Master Code (KLMC) was extended to support massive parallelism on high-performance computers.

## 1 Introduction

Manganese oxide, MnO_2_, has a history of successful application as a cathode material in lithium-ion batteries (LIBs) since its commercialization in the 1990s.^1^ Recently, MnO_2_ has gained attention for large energy storage applications, driven by the increasing market demand for energy storage systems and hybrid/electric vehicles. Manganese-based LIBs are particularly advantageous for vehicle applications due to their exceptional stability compared to nickel and cobalt based LIBs, which are prone to thermal runaway.^2,3^ Moreover, from a sustainability perspective, manganese-based cathodes provide significant benefits as they are nontoxic and abundant, making them an environmentally friendly and cost-effective alternative to traditional materials.

In common with many other materials, MnO_2_ is highly polymorphic with multiple phases only slightly separated in energy under ambient conditions.^4−9^ The polymorphic behavior leads to difficulties in the synthesis of a single partially lithiated phase and the phase transformations observed in battery charging/discharging processes.^10−13^ Moreover, this behavior has also been suggested to be responsible for the shorter than desired battery lifetimes and the subsequent efforts toward the optimization of current and the search for new cathode materials.

Among the various polymorphs of MnO_2_, λ-MnO_2_, which has the spinel structure, is widely used in LIBs as a cathode material whose three-dimensional framework structure facilitates the transport of lithium ions through the tetrahedral interstitial sites.^14,15^ Other polymorphs such as α-MnO_2_ (hollandite), R-MnO_2_ (ramsdellite), and γ-MnO_2_ (an intergrowth of pyrolusite (β) and ramsdellite) are all less common in LIBs, but are widely commercialized in Zn-MnO_2_ primary alkaline batteries.^16^ As a potential secondary LIB, however, the intergrown γ phase is particularly interesting as it stands out as the most electroactive cathode material in the LIB industry for capacity density, power density and energy-storage capability.^17−19^ The atomic structure of this intergrown γ phase includes two types of channels that can accommodate the electroactive cations: the (2 × 1) and the (1 × 1) that originate from the R and the β phases, respectively. Each channel type offers distinctive advantages, with the R phase hosting larger 2 × 1 channels that are ideal for facilitating fast lithium-ion diffusion and providing higher capacity compared to the 1 × 1 channels in the β phase. The propensity for the R phase to coexist with the β phase, which is thermodynamically more stable, within the intergrown γ structure poses challenges for the synthesis of the highly crystalline R structure.^20−22^ Nonetheless, R-MnO_2_ (RMO) holds significant potential, with theoretical capacity of 305 mAh g^–1^, which is more than twice that of the commonly utilized λ-MnO_2_ of 148 mAh g^–1^.^1,23^ Given that RMO can often be found as a part of intergrown structures in MnO_2_,^24^ it is crucial to understand its properties as a cathode, particularly in lithiation processes, for future MnO_2_ cathode engineering. To date, however, experimental, and computational studies of partially lithiated RMO are still incomplete with respect to both its structure, thermodynamic, and physical properties.

Previously, Thackeray et al. investigated the influence of the temperature of synthetic mixture on resultant structure of the lithiated RMO and its observed voltage profiles.^23^ More recently, a combined experimental and modeling study by Gupta et al. provided further insight and a mechanistic explanation of electrochemical lithiation of RMO,^25^ where their simulations employed density functional theory (DFT) and a cluster-expansion technique to sample the Li distribution in the material.^26−28^ Although the experimentally observed voltage profiles^25,29^ are qualitatively similar, there can also be remarkable differences, which are also seen on comparison with their simulation data. Computational methods prove to be insightful in studies of batteries materials as reviewed by Van der Ven et al.^30^ and Urban et al.^31^

In this study, our objective is to provide consistent insight into the structure–stability relationship, electrochemical, and thermodynamic characteristics of RMO during lithium intercalation using unbiased grand canonical Monte Carlo simulations. Our approach uses a computationally efficient interatomic potential method to overcome statistical representability issues inherent in DFT and cluster expansion-based methods, which will be discussed in Section 3. Through Monte Carlo sampling, we amassed a vast number of sample structures of RMO spanning feasible lithium concentrations within a simulation unit cell. These samples form the basis for constructing a grand canonical ensemble (GCE), allowing us to approximate the thermodynamical properties of RMO with various lithium concentrations for a range of working temperatures. In our analysis, we elucidate the mechanistic process of lithium intercalation using thermally averaged radial distribution functions (RDF) and simulated X-ray diffraction (XRD) patterns; and discuss the calculated voltage profiles and entropy changes on lithium intercalation.

## 2 Method and Computational Details

To investigate the lithiation reaction in R-MnO_2_, we first need to define the potential energy landscape, i.e., how the potential energy changes with atom positions. Due to finite computer resources, we have chosen to employ an approach based on the Born model of ionic solids and the use of analytical interatomic potentials (IPs), as we will need to evaluate (and refine) many configurations, i.e., find and explore many important local ergodic regions on several (one per concentration of lithium) potential energy landscapes. The success of this method depends on the ability to sample enough of the important regions of the landscapes and the quality of the IPs to reproduce the correct physics.

### 2.1 Interatomic Potentials

Our potential energy function is a sum of pairwise long-range Coulomb terms, as well as short-range pairwise parameter-dependent analytical functions composed of Buckingham, polynomial, and Lennard-Jones terms. These potential models are implemented in the General Utility Lattice Program (GULP) code, which also has routines for relaxing atomic structures and calculating physical properties.^32−35^

The Buckingham potential is composed of two terms
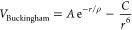
1where *r* is the interatomic distance and *A*, ρ, and *C* are the species dependent potential parameters. In this work, the Buckingham potential is employed to describe interactions between the manganese cations and the oxygen anions in the first coordination shell; and is offset in energy by a constant. Interactions between lithium cations and the oxygen anions, as well as between oxygen anions, are described by a single Buckingham function with a large distance cutoff. To ensure continuity and smoothness across the short-range distances, polynomial tapering functions are employed, which take the following form
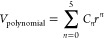
2where *n* and *C*_*n*_ are the term order and corresponding coefficient, respectively.

Additionally, to include the effect of electronic polarization, the shell model^36^ is employed, in which the ion (and its formal charge) is divided into a core and a shell (two point charges) that are connected by a harmonic spring. Taking δ as the separation distance between the core and the shell, the harmonic spring potential
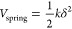
3where *k* is the spring constant, as opposed to the Coulomb interaction between the core and shell of the same ion.

GULP was used to perform geometry optimization of sample structures (structure and energy evaluation with tolerance parameters: *F*_tol_ = 10^–10^ eV, *G*_tol_ = 10^–6^ eV/Å, and *X*_tol_ = 10^–8^ Å). Note that, in contrast to Mn^3+^ and O^2–^ ions, it is assumed that a polarizable model was not necessary for Li^+^ and Mn^4+^ ions, i.e., a rigid ion model was employed: only one point charge (core only) per Li^+^ or Mn^4+^ ion.

Braithwaite et al.^37^ have already developed IP parameters for simulating LiCoMnO cathode materials with a λ-phase (spinel) MnO_2_ framework. The parameters of their IP for the Mn–O and Li–O pairwise interactions were fitted to reproduce the crystal structures of β-MnO_2_ and Li_2_O, respectively.^38,39^ However, since their potentials do not get the correct order of stability for the R and λ MnO_2_ polymorphs, we have subsequently refined the IP parameters of Braithwaite et al. after adding energy offsets and tapering polynomial functions to obtain the correct order of stability: R > λ, which aligns better with experimental observations and density functional theory calculations.^10,40−42^ The resulting set of IP parameters is summarized in Table 1. Note that the number of decimal places reported for the polynomial coefficients is kept large to ensure a smooth and continuous energy landscape, which is assumed for the geometry optimization algorithms employed here.

**Table 1:** Interatomic Potential Parameters for Modelling Lithiated R-MnO_2_

species	potential functions	parameters	*r*_min_ *– r*_max_ (Å)
Mn(IV) core–O shell	Buckingham	*A* = 3087.826 eV	0.0 – 2.0
		ρ = 0.2642 Å	
	polynomial	*C*_0_ = −1.0 eV	0.0 – 2.0
	polynomial	*C*_0_ = −91.561008 eV	2.0 – 3.0
		*C*_1_ = 313.864964 eV Å^–1^	
		*C*_2_ = −337.087774 eV Å^–2^	
		*C*_3_ = 161.755949 eV Å^–3^	
		*C*_4_ = −36.321307 eV Å^–4^	
		*C*_5_ = 3.120870 eV Å^–5^	
Mn(III) shell–O shell	Buckingham	*A* = 1686.125 eV	0.0 – 2.0
		ρ = 0.2962 Å	
		*C* = 6.0 eV Å^6^	
	Buckingham	*A* = 0 eV	2.0 – 25.0
		ρ = 1 Å	
		*C* = 6.0eV Å^6^	
	polynomial	*C*_0_ = −1.0 eV	0.0 – 2.0
	polynomial	*C*_0_ = −85.630398 eV	2.0 – 3.0
		*C*_1_ = 298.452500 eV Å^–1^	
		*C*_2_ = −319.822048 eV Å^–2^	
		*C*_3_ = 152.568677 eV Å^–3^	
		*C*_4_ = −34.034460 eV Å^–4^	
		*C*_5_ = 2.905797 eV Å^–5^	
Li core–O shell	Buckingham	*A* = 426.48 eV	0.0 – 25.0
		ρ = 0.3 Å	
O shell–O shell	Buckingham	*A* = 22.41 eV	0.0 – 25.0
		ρ = 0.6937 Å	
		*C* = 32.32 eV Å^6^	
Mn(III) core–Mn(III) shell	harmonic spring constant	*k* = 148 eV Å^–2^	0.0 – 0.8
O core–O shell	harmonic spring constant	*k* = 20.53 eV Å^–2^	0.0 – 0.8

species	atomic charges (*e*)
Mn(III) core	1.971
Mn(III) shell	1.029
Mn(IV) core	4.000
O core	0.513
O shell	–2.513
Li core	1.000

Whereas Mn^4+^ cations retain fully symmetrical configuration in octahedral environment, Mn^3+^ would undergo a well-known Jahn–Teller distortion, which is however not reproducible with simple classical interatomic potentials adopted by Braithwaite et al.^37^ We note that commonly used density functional theory approaches suffer from the same problem at the local and generalized gradient approximation levels of theory, see e.g., refs (43−45).

In Table 2, we compare the calculated lattice parameters for the R and λ phases of MnO_2_ with experimental values.^46,47^ To test the sensitivity of our results with respect to the potential parameters, four independent runs were completed after applying a 1% increase to *A*, ρ, and *C* parameters of the O_shell_–O_shell_, and the *k* parameter for the oxygen species, respectively. The largest increase to the percentage change generated to at least one of the lattice parameters of R-MnO_2_ was 0.10, 0.46, 0.02, and 0.03, respectively. Thus, results are more sensitive to the value chosen for ρ. In this case, the corresponding largest change in the nearest Mn–O bond distance, which will directly affect the radial distribution functions and X-ray diffraction patterns, is less than 0.3%.

**Table 2:** Lattice Parameters for Two Different Polymorphs of MnO_2_

	lattice constants (Å)	
structures	experiment^a^	this work (err. %)
R-MnO_2_ (*Pbnm*)	*a* = 4.532	*a* = 4.710 (+3.92)
	*b* = 9.267	*b* = 8.843 (−4.57)
	*c* = 2.864	*c* = 2.853 (−0.39)
λ-MnO_2_ (*Fd*3̅*m*)	*a* = *b* = *c* = 8.057	*a* = *b* = *c* = 8.008 (−0.60)

a Refs (46,47).

### 2.2 Modeling the Lithium Intercalation Reaction in R-MnO_2_

Consider the following lithium intercalation reaction
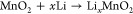
4where *x* represents the mole fraction of lithium as a value between and including 0 and 1. The reaction equation involves the reduction of manganese, Mn^4+^ to Mn^3+^, and the oxidation of lithium, Li to Li^+^. The IP method employed in this study lacks the capability to incorporate such redox reactions because of the absence of onsite electronic terms, and the IP energy is defined with respect to infinitely separated ions, which are not charge neutral. Consequently, an energy contribution from these redox reactions (redox and oxidation potentials) must be explicitly added. These potentials are crudely approximated by the ionization potentials of the respective gas species, but for more accurate calculations, environment corrections from the host materials should also be included. Here, the reaction energy in eq 4 is therefore calculated using the following formula
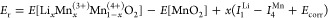
5In eq 5, the expression on the right-hand side consists of three terms: the first two terms represent the energies of RMO before and after the lithium intercalation, respectively. These two terms can be obtained from GULP calculations. The *I*_1_^Li^ and *I*_4_^Mn^ terms denote the first and fourth ionization energies of lithium and manganese atoms, respectively; and the last term, *E*_corr_, is an empirical environment parameter, accounting for the energy changes associated with the removal of a lithium atom from its source (anode) and correcting the actual reduction energy of Mn^4+^ to Mn^3+^, which occurs within RMO rather than in gas phase. In Table 3, the values of ionization energies and *E*_corr_ used in this work are provided. The correction energy term, *E*_corr_, includes the standard formation energy of lithium atom at 298.15 K, which is 1.715 eV, i.e., Li anode chemical potential vs vacuum level,^48^ and the remaining contribution of 5.676 eV is chosen to reproduce the experimentally measured voltage of a fully charged RMO with respect to the lithium electrode.^25,29^

**Table 3:** RMO Lithiation Reaction Energy Corrections^a^

parameters in eq 2	(eV)
*I*_1_^Li^	5.39171
*I*_4_^Mn^	51.2
*E*_corr_	7.391

a The first and fourth ionization energies of lithium and manganese from ref (48), and the empirical correction term used in this study, *E*_corr_, are presented.

### 2.3 Applied Monte Carlo Method

#### 2.3.1 Structural Model

**Figure 1:**
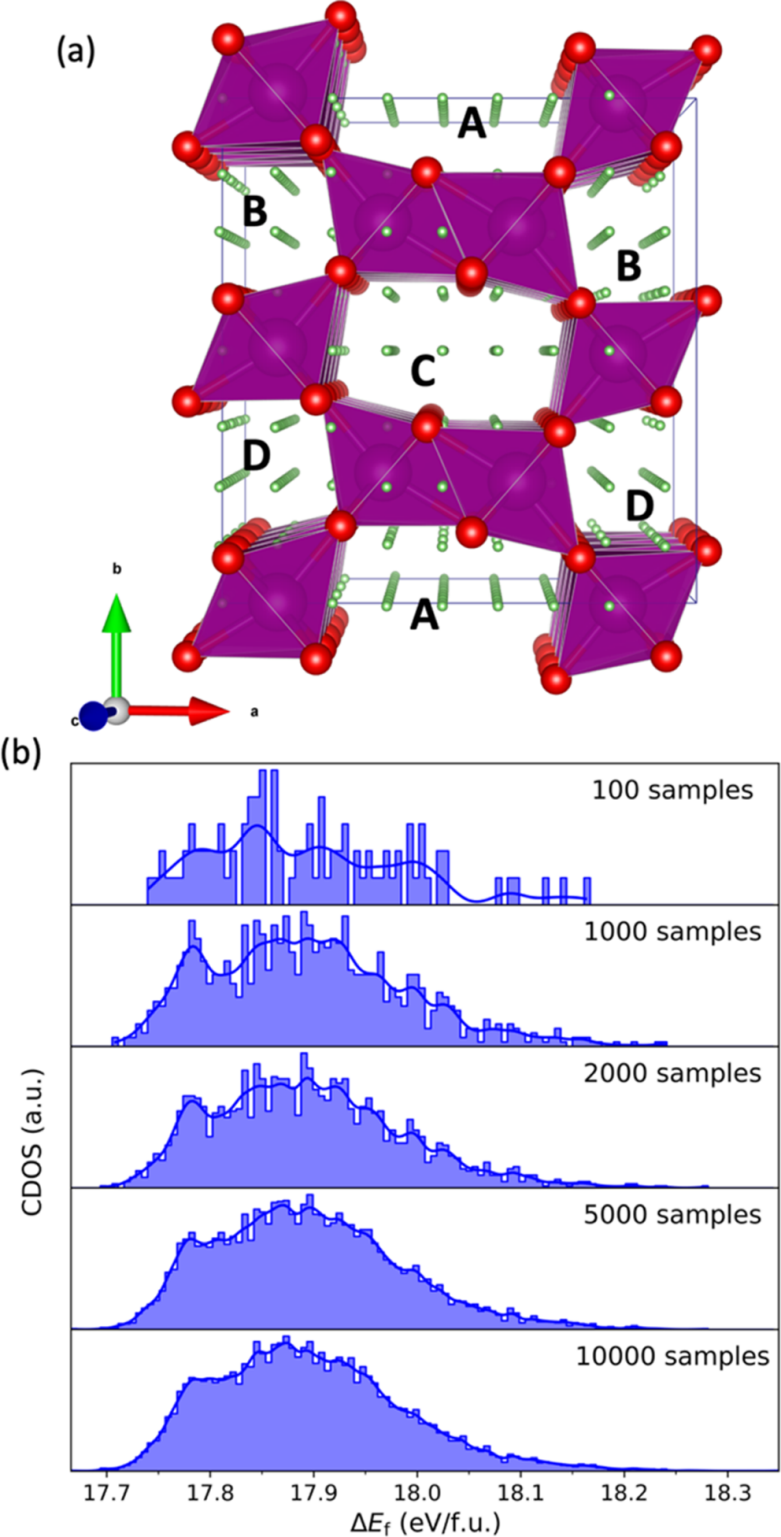
In panel (a), we present the simulation cell used throughout this project, which contains 24 MnO_2_ formula units, shown as a combined ball-and-stick and polyhedral model, where purple and red colors are reserved for Mn (within polyhedron) and O ions, respectively. The small green spheres represent the grid points where lithium ions can be initially located within the four 1 × 2 channels, labeled A to D, during the KLMC Monte Carlo sampling process. Histogram plots in panel (b) show the number of states found together with the calculated density of states curve, obtained by summing over all configurations represented by normalized Gaussian functions with the dispersion of *N*^–1/5^ where *N* is a number of samples, for Li_0.5_MnO_2_ as a function of Δ*E*_f_ = *E*(Li_0.5_MnO_2_) – *E*(MnO_2_) after evaluating 100, 1000, 2000, 5000, and 10,000 configurations (Monte Carlo sample points as described in the main text).

Ramsdellite MnO_2_ (RMO) is composed of MnO_6_ octahedra secondary building units that form infinite long planks, with a cross section of two octahedra, by edge-sharing. Each octahedron edge shares with four neighboring octahedra within a plank. Each octahedron also corner-shares to octahedrons in each of two neighboring planks so that the planks themselves construct 2 × 1 channels; see Figures 1a,3c, and 5. In this investigation, we used a 2 × 1 × 3 supercell of the conventional RMO unit cell, which contains 24 formula units of MnO_2_, as depicted in Figure 1a. The choice is made based on the available computational resources we have for this project and the desire to maximize both the supercell length in each direction and the number of configurations that can be sampled. The chosen supercell is approximately 9 Å in each direction and contains up to 96 atoms when fully lithiated with 24 lithium ions. The lithium ions are accommodated within the 2 × 1 straight channels that run along the crystallographic *c* axis—there are four such channels within the supercell—as shown in Figure 1a. With this choice, we ensure a reasonable statistical representation of intermediate lithium concentrations in RMO, i.e., that enough data points can be modeled for the mole fraction of lithium, 0 < *x* ≤ 1 in Li_*x*_MnO_2_, with possible *x* values of 1/24, 2/24···, 23/24, 1.

#### 2.3.2 Stochastic Sampling

To generate a sufficient number of configurations for each lithium concentration, we used the Monte Carlo method that is implemented within our software package, the Knowledge-Led Master Code (KLMC).^49−51^ This is in fact an extension to the KLMC module for modeling solid solutions; alternative techniques include that proposed by Allan et al.^52^ and those implemented within the SOD^53^ and DL_MONTE^54,55^ codes.

Compared to common dense crystalline solids, the RMO structure has a relatively “large” interstitial space in which we do not have *a priori* knowledge of specific “empty” sites that can be occupied by lithium ions. To probe the available interstitial space, we have defined a set of grid points evenly spaced at ca. 1.03 Å apart within this region. This was achieved by first defining a 9 × 9 × 9 uniform grid across the supercell (simulation box) as depicted in Figure 1a. Then we checked the suitability of each grid point by comparing its proximity to occupied lattice sites. All grid points within 1 Å from a manganese or oxygen site were then purged, resulting in 360 interstitial sites. During the sampling process, lithium ions were randomly placed on this reduced grid, while concurrently an equal number of the Mn^4+^ cations, randomly selected, were replaced with Mn^3+^ cations to ensure charge neutrality of the system.

**Figure 2:**
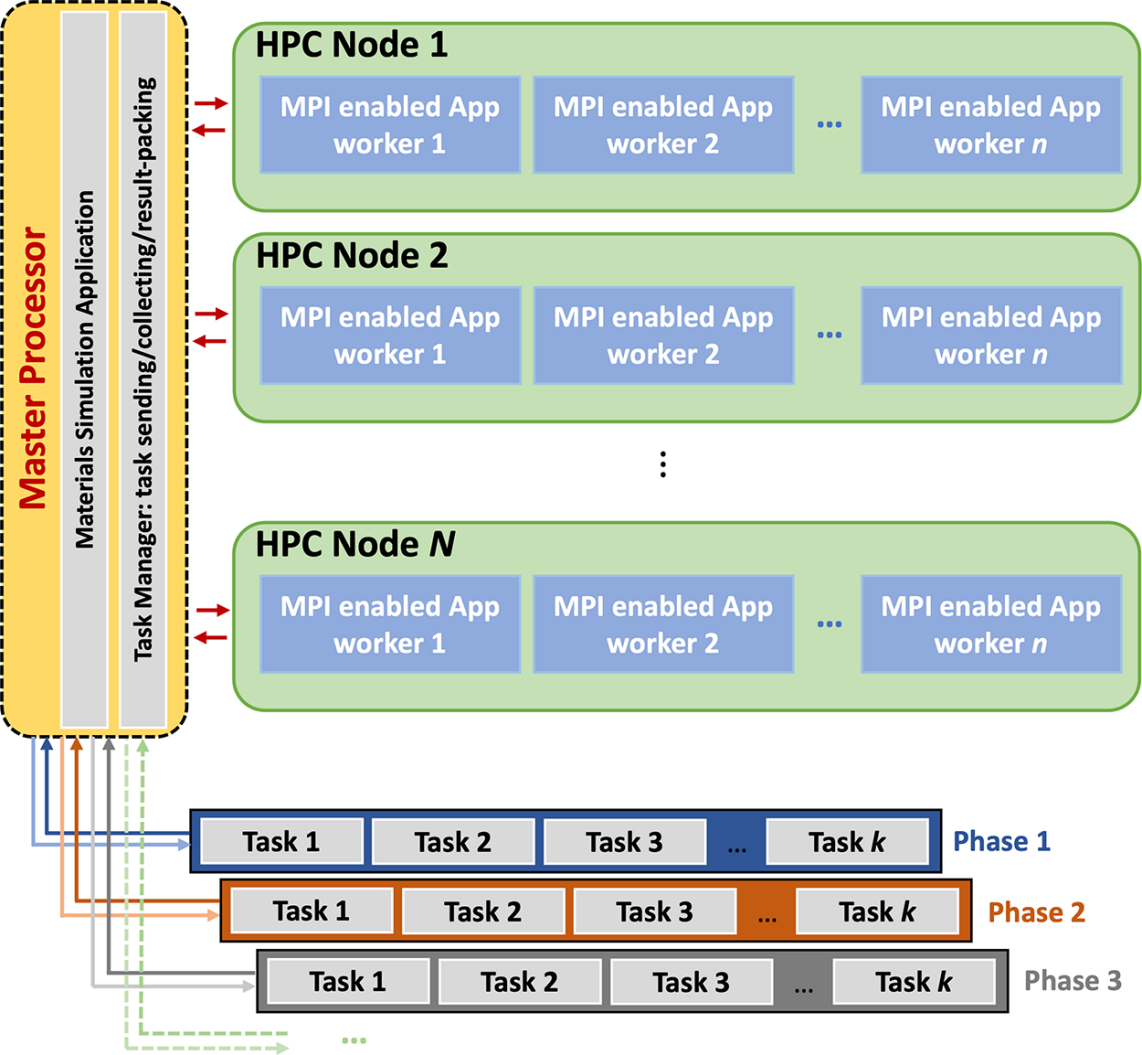
High-performance computing extension of KLMC, utilizing the “master-worker” architecture where the GULP software is used as the materials simulation application, executed on a group of CPUs referred to as “workers” with support of Message-Passing Interface (MPI). The diagram describes how the code uses number *N* HPC nodes, each node consisting of number *n* workers. In our implementation, one CPU is responsible for managing tasks (i.e., GULP calculations), including stochastic samplings; distribution of tasks over workers across HPC nodes; and collection of results for further analysis. The bottom part of the diagram shows the three-phase optimization process employed in this study, i.e., how the KLMC code performs multiple energy landscape evaluations.

For each of the possible 24 lithium concentrations (i.e., ignoring *x* = 0, or the fully delithiated RMO) we have used KLMC to generate 20,000 random structures. These structures then underwent a three-phase optimization process to minimize the potential energy using a newly developed parallel version of KLMC that is designed to exploit high-performance computers (HPC). The parallelization of the KLMC software is summarized in Figure 2. In the first phase, while employing a rigid-ion model and fixed lattice parameters, the atomic coordinates were optimized. In the second phase, the rigid ion model was replaced by the polarizable shell model (shells added to Mn^3+^ and O^2–^ ions) and the positions of all cores and shells were refined while still maintaining constant lattice parameters. In the third and final phase, the model was fully relaxed under constant (zero) pressure (fractional coordinates and lattice parameters optimized). This multiphase optimization approach is adopted to avoid problems inherent in the implementation of the shell model within GULP, which assumes atoms start in physically sensible positions, whereas here we have an element of randomness in the initial atomic structure. Strong electric fields can result from ions approaching each other too closely, particularly in the initial line optimization steps, leading to the unwanted breaking of the spring between a core and shell (δ > 0.8 Å).

Following the multiphase optimization process, structures with imaginary vibrational frequencies at the γ point were discarded. From the remaining structures, we selected the 10,000 lowest energy structures to represent each of the 24 concentrations. The cap of just 10,000 structures is sufficient to ensure key configurations are found for our chosen simulation cell, as shown in Figure 1b where good convergence is already achieved with 5000 samples for Li_0.5_MnO_2_, the system which has, combinatorially, the largest number of possible initial lithium configurations in the chosen RMO simulation cell.

There are other types of sampling approaches, such as the cluster expansion method^25−28,56,57^ or the inversion method^58^ that can be combined with *ab initio* calculations to predict the stability of a cathode material in the lithiation process. These approaches rely on the effective configurational Hamiltonian model or the atom–atom interactions in the context of an Ising-model-like predefined lattice.^30,59,60^ We have chosen a Monte Carlo approach that has the advantage that we can include the many-body correlations, i.e., long-range Coulomb interactions, and, as a result, the generated X-ray diffraction patterns are more accurate. Nonetheless, the Monte Carlo approach has its own disadvantage in that it suffers from finite-size effects, which is particularly detrimental to our ability to model the infinite dilute limit. To address this case, we have employed an embedded cluster technique, as now described below.

#### 2.3.3 Infinite Dilution Limit

We used the Mott–Littleton approach^61^ as implemented in GULP to investigate point defects in the seven lowest energy local minima found for Li_1/24_MnO_2_, i.e., consider the limit of infinite dilution–see Figure S1 in Supporting Information (SI). This approach guarantees convergence of relevant thermodynamic properties (probing the microstates within the energy range up to 7 *k*_B_*T* from the ground state).

In the Mott–Littleton method, the origin of the model (doped RMO lattice) is chosen to be at the center of the defect from which three regions are defined: region I, region IIa, and region IIb, using two radial cutoff values. In region I, a pair of defects consisting of a lithium interstitial impurity and a reduced Mn ion considered as an impurity is placed at the lattice positions found using the supercell method introduced above, and all ions in region I are explicitly relaxed with respect to their atomic positions. Note that the approach can model separately both a lithium interstitial impurity and a reduced Mn ion, however, our supercell results indicate that the distance between these point defects is minimized. Relaxation of ions in region IIa due to the defect in region I is modeled within a linear response approximation. Polarization of ions in region IIb that extends to infinity is also calculated using a linear response approximation without including the short-range interaction with ions in region I. The difference between region IIa and region I cutoff radii is chosen to be at least equal to the short-range potential cutoff. In contrast to the supercell approach, the reaction energy of inserting a single lithium-ion dopant into the infinite RMO host is defined as
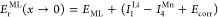
6where *E*_ML_ is the calculated Mott–Littleton energy (the energy difference between the perfect and defective systems), and the other terms, *I*_1_^Li^, *I*_4_^Mn^, and *E*_corr_, are defined earlier and used in eq 5. The energy of reaction eq 4 at this limit is therefore *x* × *E*_r_^ML^, where *x* represents the mole fraction of lithium in Li_*x*_MnO_2_.

In our Mott–Littleton defect calculations, we chose the defect center to lie midway between respective initial positions of the Li^+^ dopant and the reduced Mn^3+^ ion. A region I cutoff of 25 Å and a region II cutoff of 50 Å were used to ensure convergence (with respect to radii cutoffs) of defect energies, *E*_ML_, to 0.01 eV; see Figure S2 in Supporting Information (SI). Vibrational analyses confirmed the local stability of each defect pair, i.e., no imaginary frequencies were found.

In our statistical analysis of the lithium distribution, to obtain the configurational entropy contributions we also need to evaluate the degeneracy, or multiplicity of relevant microstates. For the infinite dilution limit, the multiplicity of the structures was calculated by applying the symmetry operators of pure RMO (space group *Pbnm*) to the seven structures used in Mott-Littleton calculations. Of the resulting structures, the ML model was applied only once for each symmetry inequivalent.^62^ The calculated ML defect energies along with the reaction energies are shown in Table 4 in the Results and Discussion section.

**Table 4:** Lithium Intercalation Energies in Li_*x*_MnO_2_ Ramsdellite at the Limit of Infinite Dilution^a^

rank	*E*_ML_ (eV)	*E*_r_^ML^ (eV)	*E*_r_ (eV)	degeneracy
1	35.4761	–2.9412	–2.9984	2
2	35.4232	–2.9940	–2.9949	1
3	35.5146	–2.9027	–2.9278	2
4	35.5577	–2.8596	–2.8803	1
5	35.6512	–2.7661	–2.8558	2
6	35.7045	–2.7128	–2.8511	1
7	35.5146	–2.9027	–2.8172	1

a Reaction energies calculated using the Mott–Littleton (*E*_r_^ML^) approach are compared to the results of periodic supercell (*E*_r_) calculations for the seven top-ranking configurations found by Monte Carlo sampling of lithium distribution in LiMn_24_O_48_ simulation cell. *E*_ML_ is the Mott–Littleton defect energy obtained using GULP.

### 2.4 Thermodynamical Analysis

The partition function of the (*x*PT) canonical ensemble (CE) and the (μPT) grand canonical ensemble (GCE) are used to compute thermodynamical properties.^30,63,64^

#### 2.4.1 Grand Canonical Ensemble

Denoting μ, *k*_B_, and *T* as the chemical potential, the Boltzmann constant, and the temperature, the grand partition function *Z* can be approximated as
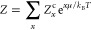
7where the summation is over the 25 lithium concentrations of the selected RMO system and the canonical partition function for each lithium concentration is denoted by *Z*_*x*_^c^. Here, μ is referenced to the chemical potential of the lithium anode, i.e., μ = μ^cathode^ – μ^anode^, as accounted for by the *E*_corr_ term in eq 5. In our simulations, μ is the energy cost of adding *x* lithium to RMO per formular unit, using lithium anode as the source. The canonical partition function of this system is defined as
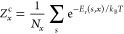
8where *s* labels each sample point (fully relaxed configuration), *N*_*x*_ is the number of sample points and the summation is over all (typically, 10,000) sample points for a given lithium molar fraction, *x*. *E*_r_(*s*, *x*) is the reaction energy of lithium intercalation as defined by eq 5. Note that in the case of infinite dilution limit, i.e., *x* → 0, the Mott–Littleton derived reaction energies, *E*_r_^ML^ in eq 6, were scaled to match the size of the supercell used in the Monte Carlo simulations, i.e., by a factor of 24.

#### 2.4.2 Observables

The averaged observable property *f* for the GCE of samples *s* and lithium concentrations *x* is given by
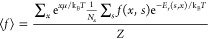
9

For example, for a given temperature *T* and chemical potential μ, a GCE averaged lithium concentration, ⟨*x*⟩, can be calculated as follows
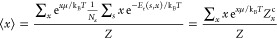
10

This equation can be rewritten in a compact form
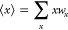
11where *w*_*x*_ is the mole fraction distribution function
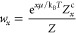
12

#### 2.4.3 Structure and Crystallographic Data

The method outlined above was further applied to calculate the average radial distribution functions and simulated X-ray diffraction (XRD) patterns of Li_*x*_MnO_2_. For the generation of radial distribution functions and XRD patterns, the Python packages from refs (65,66) were used, respectively. Each pair distance from each sample entering the RDF is represented by a normalized Gaussian function with the dispersion of 0.06 Å (i.e., a temperature-independent parameter as the RDF simulation here does not include broadening due to atomic vibrations). For the XRD simulations, all calculations were performed for the X-ray wavelength of 1.54059 Å, corresponding to the Cu Kα radiation in an experiment.

#### 2.4.4 Voltage Profile

Following the Nernst thermodynamic model of electrochemical cells, the model RMO voltage at a certain observed lithium concentration ⟨*x*⟩ is given as^30^
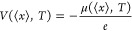
13However, for a given temperature *T*, according to eqs 11 and 12, ⟨*x*⟩ is obtained as a function of μ. Therefore, it is beneficial to derive an expression for μ as a function of the lithium concentration ⟨*x*⟩, as required by eq 13. The inversion of eq 10 is performed numerically: we define a dense grid of μ from −7.5 to +5.0 eV—evenly spaced with an interval of 2.5 × 10^–5^ eV—to map the corresponding observed lithium concentration ⟨*x*⟩ spanning the full stoichiometric range and ensuring that any selected value of *x* is bracketed by precalculated values of ⟨*x*⟩ within a 10^–5^ error.

#### 2.4.5 Gibbs Free Energy and Entropy

As the chemical potential of RMO is related to the Gibbs free energy, *G*_r_, of the lithium intercalation reaction
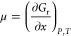
14the Gibbs free energy change from the fully delithiated electrode referenced to the anode at the respective lithium molar fraction *x* is
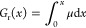
15Here, we compute the associated changes in the free energy by using the following thermodynamic relation
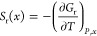
16where *S*_r_ is a change in the entropy of before and after lithium intercalation reaction. In practice, the free energy integral in eq 15 is calculated numerically using μ(⟨*x*⟩) obtained earlier. Finally, the Gibbs free energy is numerically differentiated with respect to *T* yielding the entropy referenced to the anode using two-point central differences with Δ*T* = 0.5 K.

## 3 Results and Discussion

### 3.1 Infinite Dilution Limit

In Table 4, we summarize the results of Mott–Littleton calculations and compare them to the energies obtained from the supercell Monte Carlo simulations.

We find a close match in reaction energies between the two methods. The Mott-Littleton reaction energies, *E*_r_^ML^, are consistently higher (reaction slightly less favorable) compared to their supercell counterparts except for the configuration ranked seventh that relaxed to a symmetry equivalent local minimum of that found for configuration ranked third; see Figure S1(3),(7) in Supporting Information (SI). There is also a subtle change in ranking between the closely lying first and second energy minima.

### 3.2 Lithium Distribution

**Figure 3:**
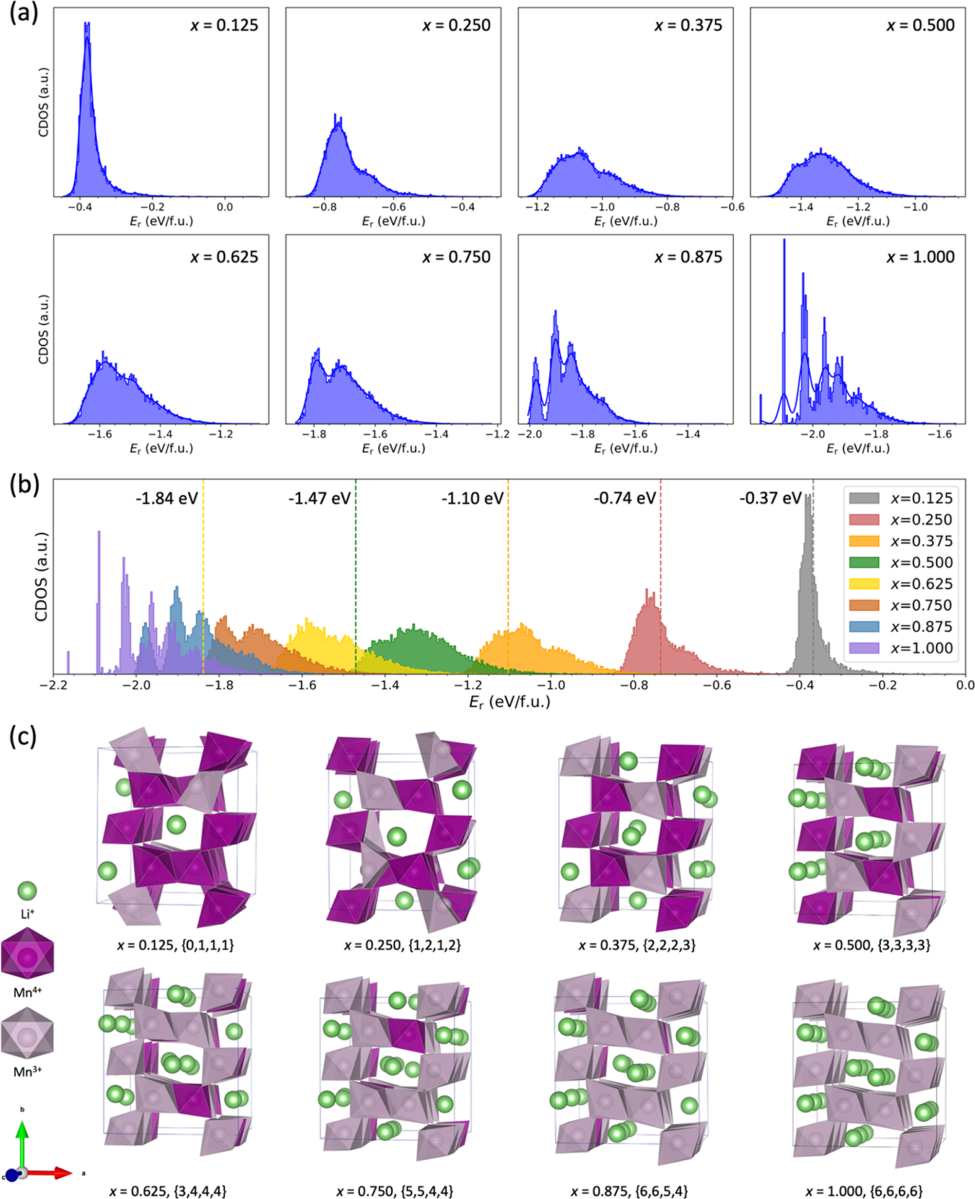
Histogram plots in panel (a) show the number of states found together with the calculated configurational density of states curve, obtained by summing over all configurations represented by normalized Gaussian functions with the dispersion of 0.158 eV, for selected (8 of the 24) systems of Li_*x*_MnO_2_ as a function of *E*_r_. The energy range in each plot is chosen to magnify its respective details. The CDOS plots are combined into a single graph in panel (b), for an easier comparison of the energy shifts, where the colored dashed lines mark *x* × *E*_r_^ML^. The structures in panel (c), shown as the ball and polyhedral models, are the global minimum structures of each selected Li_*x*_MnO_2_ system. Note that O ions occupy the vertices of each polyhedron and are not shown as spheres to improve clarity in visualizing the positions of Li^+^ ions. Numbers within the curly braces, {A, B, C, D}, indicate the number of Li^+^ in the four 2 × 1 channels, labeled A to D in Figure 1.

Stochastic sampling of the lithium ions over the 360 interstitial sites (the grid described above) and the substitution of Mn^3+^ ions on the 24 Mn^4+^ sites has produced a series of canonical ensembles for Li^+^/Mn^3+^ distributions, with their respective configurational densities of states (CDOS) shown in Figure 3a along with the tentative global minimum (GM) structures for selected lithium concentrations given in Figure 3c. As the lithium concentration increases, there is a shift in the CDOS to lower energies, which correlates to that found in the limit of infinite dilution (as shown in the previous section) where the distribution will collapse to a delta function at *x* = 0. As a zero approximation of noninteracting defects, the calculated *E*_r_^ML^ for the global minimum provides useful estimates of the shift in CDOS with *x* in Figure 3a. For example, the estimated *E*_r_ for *x* = 0.125, 0.250, 0.375, and 0.500 are −0.37, −0.74, −1.10, and −1.47 eV–see the dashed lines in Figure 3b–which are similar to the lowest CDOS peaks at −0.38, −0.76, −1.13, and −1.43 eV that are obtained from the Monte Carlo simulations. This unexpected good agreement, however, breaks at higher values of *x*. For example, the predicted value at *x* = 0.625 is −1.84 eV, whereas the lowest CDOS peak is found at −1.58 eV.

**Figure 4:**
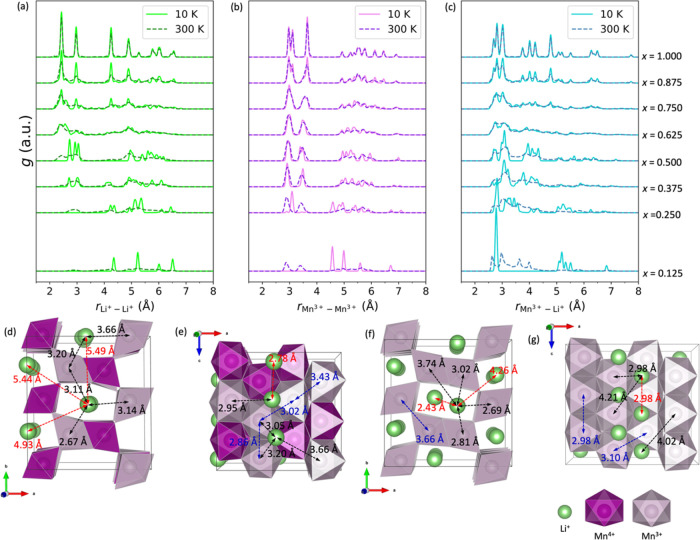
GCE averaged radial distribution functions, *g*, as a function of distance: *r* = *r*_Li^+^–Li^+^_, *r*_Mn^3+^–Mn^3+^_, and *r*_Mn^3+^–Li^+^_ in Li_*x*_MnO_2_ for different values of μ and at two different temperatures, 10 and 300 K. The second lowest minimum structure of Li_0.5_MnO_2_ is in (d, e), and the global minimum structure of LiMnO_2_ is in (f, g) where purple and gray spheres within polyhedra represent Mn^4+^ and Mn^3+^, respectively; O atoms are at the vertices of polyhedra, which are omitted for clarity; and green spheres represents Li^+^. The arrows in red, blue, and black colors show the interatomic distances of Li^+^–Li^+^, Mn^3+^–Mn^3+^, and Mn^3+^–Li^+^ respectively.

Within these GM structures, the lithium ions tend to distribute evenly across available interstitial positions in the 2 × 1 RMO channels. For example, the GM structure for *x* = 0.5 has evenly spaced Li ions aligned along one column in each channel (typically, to find this GM structure with the observed specific alternating Mn^3+^/Mn^4+^ pattern—see Figure 3c where *x* = 0.500—more than 20,000 Monte Carlo samplings is required, which is not really surprising, considering the largest search space at this lithium concentration), whereas the GM structure for *x* = 1 has two such columns of lithium ions per channel. One exception is observed for the *x* = 0.875 (or 21/24) GM structure, where at least 5 lithium ions can be placed in each channel. The {6,5,5,5} configuration is one of four equivalent structures with the most even lithium distribution but is found to be 0.001 eV higher in energy than the {6,6,5,4} GM structure shown in Figure 3c where *x* = 0.875. This observation aligns with the expectation that the most stable structures adopt configurations with lithium ions and lithium vacancies (in fully lithiated channels) distributed homogeneously across available sites, thus minimizing electrostatic repulsion between positively charged lithium ions (and negatively charged vacancies, respectively). Indeed, the distribution of Mn^3+^/Mn^4+^ also influences the energy; however, this effect is less than that due to the distribution of lithium ions. This is evidenced by the small energy difference of 0.01 eV between the GM and the second lowest minimum structures for *x* = 0.5, (see the respective structures in Figure 3c where *x* = 0.500 and Figure 4d,e), where both structures have the same distribution of {3,3,3,3} lithium ions, while the GM structure has a pattern of alternating Mn^3+^/Mn^4+^ configuration.

In the first four CDOS plots in Figure 3a, corresponding to low lithium concentrations (*x* ≤ 0.5), *E*_r_ decreases while the spread in the distribution appears to increase with increasing *x* as there is an increase in the relative heights of the higher energy peaks compared to the first peak. In particular, at *x* = 0.125 and 0.250, there are some incomplete octahedra formed by Mn^3+^/Mn^4+^. This observation is attributed to a severely distorted shell on (or polarization of) Mn^3+^ ions induced by a high local electric field in the presence of adjacent lithium ions, which disappears at higher concentrations where lithium ions start getting more evenly distributed into the 2 × 1 channels. As the lithium concentration increases from 0.625 to 1.000, *E*_r_ continues to decrease, but now the peaks start to separate. The additional peaks can be attributed to the increased energy cost of moving a lithium ion from a channel containing two columns of lithium ions to a channel that is already occupied with two columns of lithium ions. For example, at *x* = 1, the first peak at −2.16 eV corresponds to the GM structure shown in Figure 3c of *x* = 1.000, where all channels are equally occupied with a lithium ion distribution of {6,6,6,6}. The second peak at −2.10 eV in the CDOS has structures with two channels containing six lithium ions and the other two channels containing five and seven lithium ions. For example, the {7,5,6,6} lithium ion distribution, which has one more/less lithium in the A/B channel than the {6,6,6,6} GM structure. The third peak at −2.07 eV includes lithium distributions that can be constructed by moving two lithium ions between channels in the {6,6,6,6} GM structure to create lithium distributions such as {7,7,5,5} or {8,4,6,6}. The lithium ion distribution of {8,7,5,4} will therefore contribute to the fourth peak, as three lithium ions are moved in the GM structure. The high-energy structures include at least one of the channels accommodating an excessive lithium ion, leading to an abrupt rise in electrostatic repulsion increasing the energy. At low lithium concentrations, the energy cost of forming such inhomogeneous lithium distributions is lower, because the channels still have enough space or vacancies to accommodate additional lithium ions. Furthermore, the variation in Mn^3+^/Mn^4+^ distribution (whose effect is less than the lithium ion distribution) also broadens the CDOS. Overall, this behavior accounts for the change in the nodality of the CDOS, from a single peak to a multinodal distribution as seen in Figure 3b.

### 3.3 Radial Distribution Function

For further analysis we have plotted in Figure 4a–c the radial distribution functions (RDF) of Li^+^–Li^+^, Mn^3+^–Mn^3+^, and Mn^3+^–Li^+^, which were obtained by averaging over the GCE at *T* = 10 K (low temperature) and *T* = 300 K (room temperature) for chosen values of the chemical potential that generate the targeted values of lithium concentration.

At 10 K and the lowest targeted lithium concentration illustrated in Figure 4, *x* = 0.125, no RDF peaks for Li^+^–Li^+^ or Mn^3+^–Mn^3+^ pairs are observed within 4 Å. Given the distance between neighboring Mn sites is less than 3.6 Å and the periodicity of our simulation cell is roughly 9 Å, the lithium ions and likewise the extra electrons on the Mn sites are as far apart as possible; the most diffuse distribution of each is predicted. In contrast, the RDF for Mn^3+^–Li^+^ shows a strong peak around 2.8 Å, suggesting that the reduction of Mn^4+^ occurs near Li^+^, which maximizes the energy of the electrostatic attraction between the two opposite charges while maintaining local charge neutrality. Alternatively, this Mn^3+^–Li^+^ pairing behavior can be explained by considering that the Madelung potential is driving a +4 charge at each Mn site, so each Li^+^ will be attracted to each Mn^3+^. At 300 K, a very small broad Li^+^–Li^+^ peak at ∼2.8 Å appears (cf. 10 K) and a well-defined Mn^3+^–Mn^3+^ peak appears at ∼2.9 and at ∼3.4 Å as higher energy structures become accessible (statistically significant). The peaks in the Li^+^–Li^+^ RDF are typically broader than those in the Mn^3+^–Mn^3+^ because the curvature of the potential for lithium displacements is much flatter than for manganese displacements; this difference is expected to increase if we had included vibrational contributions as the lithium ions are lighter and more mobile. Comparing the appearance of the peaks for short distances, the relative heights (taller peaks for Mn^3+^–Mn^3+^) indicate that there is a stronger penalty for two Li ions to approach each other within a channel than between two Mn^3+^ ions. Thus, there is a more efficient screening in the manganese oxide framework compared to along the channels that the lithium ions occupy, caused by the location of the oxygen ions. Indeed, on examination, the short Li^+^–Li^+^ separation distance is only possible when three Li^+^ ions occupy the same RMO 2 × 1 channel, an example of which for a higher Li concentration is provided in Figure 4e. Returning to the low concentration example, the tall low temperature single Mn^3+^–Li^+^ peak now splits into four peaks spread out over 2.7 to 4.0 Å as multiple statistically significant configurations include pairs of Li^+^ ions that occupy the same channel while Mn^3+^ ions mediate their repulsion. Thus, the long distance (above 5 Å) Mn^3+^–Li^+^ RDF peaks observed at 10 K decrease in height with temperature.

As lithiation proceeds to *x* = 0.250 for the lower temperature (*T* = 10 K), Li^+^–Li^+^ peaks appear between 4.0 and 4.5 Å. Considering the lowest energy Li^+^ configuration, for example, the {1,2,1,2} shown in Figure 3c where *x* = 0.250, it is clear that these peaks correspond to a pair of Li^+^ ions occupying the same 2 × 1 channel, whereas the RDF peaks near 5.0 Å and higher appear from Li^+^ ions in different 2 × 1 channels as demonstrated in Figure 4d where distances are marked using red colored arrows. For the Mn^3+^–Mn^3+^ RDF, a strong single peak appears at ∼3.1 Å, as opposed to two separate peaks at ∼2.9 and ∼3.4 Å that appeared for the lower concentration of *x* = 0.125 when the temperature was increased to 300 K. The peak near 3.1 Å corresponds to the interatomic distance of 3.02 Å between the manganese atoms within two edge-sharing MnO_6_ octahedra that is indicated in Figure 4e by a blue arrow. There is still a strong, although reduced in height, Mn^3+^–Li^+^ peak at 2.8 Å, which is accompanied by a series of dispersed smaller peaks at longer Mn^3+^–Li^+^ distances that, similar to previous analysis, can be attributed to the stabilization of a Li^+^ ion pair occupying the same channel. At the higher temperature of 300 K, we observe a clear Li^+^–Li^+^ dispersed peak at ∼2.8 Å as three or more Li^+^ ions can now occupy one channel. In the Mn^3+^–Mn^3+^ RDF, temperature splits the single peak at ∼3.1 Å into two peaks that have a larger amplitude but the same location as that previously seen for the lower lithium concentration (*x* = 0.125) and high temperature (300 K) system. These two peaks correspond to the interatomic distances of 2.86 Å parallel to the direction of the channels and 3.43 Å that are marked using blue arrows in Figure 4e between centers of edge- and corner-sharing MnO_6_ octahedra, respectively. The Mn^3+^–Mn^3+^ RDF pattern above 4.0 Å no longer has clear sharp peaks, suggesting no longer-range ordering of Mn^3+^ ions. The magnitude of the 2.8 Å Mn^3+^–Li^+^ peak is notably reduced with temperature, which we attribute to the delocalization of Mn^3+^ ions as strong local Mn^3+^–Li^+^ pairs predissociate, and the growth of a second peak at 3.0 Å is observed.

**Figure 5:**
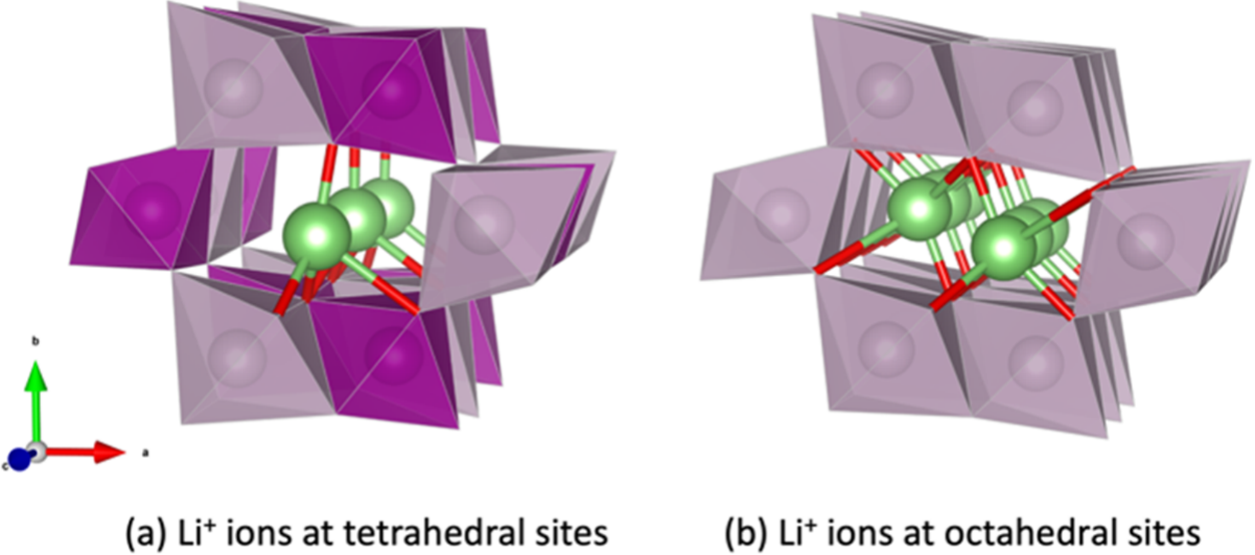
Two types of lithium intercalation sites in 2 × 1 channels of R-MnO_2_ are presented. The structures are global minima of Li_*x*_MnO_2_ where (a) *x* = 0.5 and (b) *x* = 1; sticks in green and red colors represent bonds of Li–O, and the others are as in Figures 3 and 4.

On further lithiation to *x* = 0.375 and 0.500, notable peaks in the low temperature Li^+^–Li^+^ RDF appear between 2.8 and 3.1 Å, which is attributed to the increased number of Li^+^ ions per channel; compare plots in Figure 3c from *x* = 0.250 to *x* = 0.500, which have respective Li^+^ orderings of {1,2,1,2}, {2,2,2,3}, and {3,3,3,3}. There is also the disappearance of Li^+^–Li^+^ peaks between 3.9 and 4.5 Å, as *x* increases from 0.375 to 0.500. The latter peaks occur when a channel accommodates fewer than three Li^+^ ions, as observed earlier with {1,2,1,2} for *x* = 0.250 and {2,2,2,3} for *x* = 0.375. Given the range of the peaks that appear and those that disappear are roughly one-half and one-third of the length of the simulation box used here, this suggests a strong preference for the lithium ions to maximize the distance between them as opposed to there being only three lithium sites along a 2 × 1 channel. For a higher temperature, the first Li^+^–Li^+^ peak becomes binodal at *x* = 0.500, with the appearance of two separate peaks: one at ∼2.4 Å and another at ∼3.0 Å. The Li^+^–Li^+^ peak at 2.4 Å suggests an imbalance in the distribution of Li^+^ ions across the channels, possibly resulting from such an arrangement as {4,2,3,3}, in which one channel is overpopulated. Furthermore, the channel, occupied by just two Li^+^ ions, gives rise to very low contributions to the RDF between 3.9 and 4.5 Å. Considering the Mn^3+^–Li^+^ RDFs, the 2.8 Å peak, due to strongly bound local Mn^3+^–Li^+^ pairs, is significantly reduced and broadened, regardless of temperature. At these Li^+^ concentrations, the number of Mn^3+^ ions becomes sufficient to be evenly distributed across the system and could provide adequate electrostatic screening, which is further supported by the propensity of Li^+^ ions to occupy central positions along the 2 × 1 channels, as illustrated in Figure 3c where *x* = 0.500 or Figure 4d. At *x* = 0.500, these central positions correspond to the tetrahedral sites, as shown in Figure 5a. This observation is consistent with experimental findings and other computational studies indicating that Li^+^ ions prefer energetically stable tetrahedral sites in various polymorphs of MnO_2_, rather than occupying less stable octahedral sites as depicted in Figure 5b, which will be further discussed below.^13,25,29,67,68^

At high Li^+^ concentrations, going from *x* = 0.625 to 1.000, RDF peaks progressively converge to those seen for *x* = 1.000. Notably, the 2.43 Å Li^+^–Li^+^ interatomic distance shown in Figure 4f by a red arrow is associated with a peak in the RDF that is consistently present across these concentrations, regardless of temperature. Moreover, this peak is also present for the high temperature example when *x* = 0.500 and its presence indicates that the channels with more than three Li^+^ ions become statistically significant. Another clear difference from the *x* = 0.500 distribution is the emergence of a new peak between 4.00 and 4.50 Å, due to the stabilization of Li^+^–Li^+^ pairs across adjacent channels (see the marked interatomic distance of 4.26 Å in Figure 4f). Now consider the Mn^3+^–Mn^3+^ RDFs, where the peak at ∼3.5 Å gradually shifts to the right, which describes the stretching of Mn^3+^–Mn^3+^ distance between corner-sharing manganese octahedra, as depicted in Figure 4e,f (3.43 and 3.66 Å peaks, marked with blue arrows). The stretching is accompanied by a lattice expansion, for example, a ∼22% predicted volume increase for *x* = 1.000 both from our low and room temperature simulations. Unlike the behavior seen for low lithium concentrations, *x* ≤ 0.5, where channels have sufficient space to accommodate Li^+^ ions at tetrahedral sites, at higher concentrations, *x* > 0.5, Li^+^ ions start to wedge into channels, shifting each other from the tetrahedral to octahedral sites—compare Figure 5a,b. Each channel has twice as many octahedral sites compared to tetrahedral sites. The tetrahedral sites reside between two pairs of octahedral sites, and Li^+^ ions form a staggered pattern when a channel is fully occupied at *x* = 1.000, as shown in Figure 4f,g.

Finally, at *x* = 1.000, we observe pronounced sharp peaks for all RDF patterns shown in Figure 4a–c, regardless of temperature, which indicates that the structure of fully lithiated RMO is highly crystalline and less affected by temperature variations. This finding is also consistent with the CDOS shown in Figure 3a where *x* = 1.000. As discussed earlier, there is a significant energy gap between the global minimum structure and the next lowest energy local minimum configuration. As we can see from the RDFs at 300 K, the second local minimum in the CDOS is not thermally accessible due to the high energy cost associated with adding excessive Li^+^ ions into the already fully occupied channels, which is also supported by the absence of Li^+^–Li^+^ RDF peaks at distances less than 2.4 Å in Figure 4a at *x* = 1.000. The corresponding structures, including the first and second coordination shells and respective interatomic distances are shown in Figure 4f,g. For instance, the first three Li^+^–Li^+^ peaks are observed at 2.43, 2.98, and 4.26 Å, as indicated by the red arrows.

### 3.4 X-ray Diffraction Patterns of Lithiated R-MnO_2_

**Figure 6:**
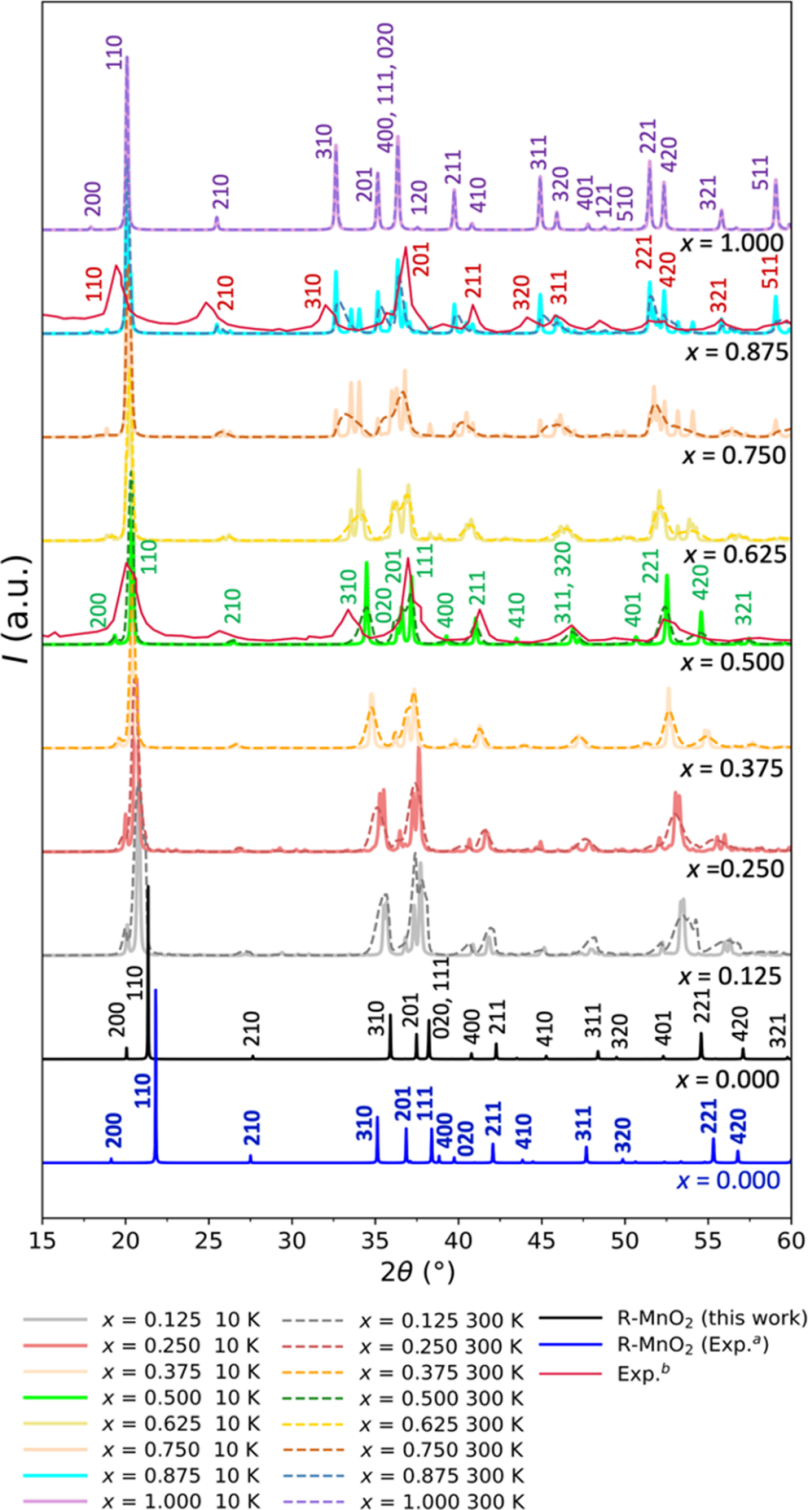
GCE averaged XRD patterns of Li_*x*_MnO_2_ (0 < *x*) at two different temperatures, 10 K (solid lines) and 300 K (dashed lines) and eight targeted lithium concentrations (different colored lines) that is obtained by fixing the appropriate value for the chemical potential; at the bottom, solid black and solid red lines are simulated XRD of pure R-MnO_2_ structure by this work and experiment ^a^ref (46), respectively; and for *x* = 0.500 and 0.875, the thinner red lines have been added that represent experimentally observed XRD patterns for the respective lithium concentrations, taken from ^b^ref (13).

The GCE-averaged X-ray diffraction (XRD) patterns are readily generated for the crystal structures obtained from the Monte Carlo supercell sampling. In Figure 6, we present XRD patterns for different Li^+^ concentrations, *x*, for both a low temperature (10 K) and room temperature (300 K). At the bottom of Figure 6, for reference, we present simulated XRD patterns of the pure RMO structure (*x* = 0.000), generated in this work along with experimental data taken from ref (46), where we observe a subtle difference. Across the given range of values considered for two-theta, the deviation from the experimental crystal structure is approximately less than 2°. This difference arises from inaccuracies inherent in the IP model employed in this work; see the comparison of the calculated and available experimental structure parameters shown in Table 2.

Generally, lithiation shifts the XRD peaks to the left; in particular, the [110] diffraction peak shifts strongly from 21.4° at *x* = 0.000 to 20.4°at *x* = 0.500 and then only weakly to 20.2° at *x* = 1.000, which agrees with experiments as lithiated and also protonated samples of RMO are reported to have an expanded lattice.^69−71^ Furthermore, at *x* = 0.500 and 0.875, the simulated XRD pattern is in good agreement with the experimental data^13^ and is shown in Figure 6 using thin red lines. Compared to this experimental data, we can identify unique peaks for [311] and [320] for *x* = 0.000, which, on lithiation, overlap for *x* = 0.500, agreeing with experiment. This observation correlates with the tendency for Li^+^ and charge-compensating Mn^3+^ ions to adopt a homogeneous distribution, which is first fully realized at *x* = 0.500 where in the ground state Li^+^ ions are distributed uniformly over channels accompanied by alternating Mn^3+^/Mn^4+^ in the framework of octahedra, which has also been verified by the RDF analysis above. On further lithiation (*x* = 0.875 and 1.000), we can again identify unique peaks for [311] and [320]. We found that the order of our calculated [311] and [320] peaks are different to that reported in experiment (cf. the experimental XRD pattern has these flipped for *x* = 0.875; see thin red line in Figure 6), and this difference may be attributed to the inherent inaccuracy in the employed IP model. Further agreement with the experiment includes the changes in the [321] and [511] peaks, which have very weak intensities at *x* = 0.500 and progressively increase in strength when further lithiated (to *x* = 0.875 and then *x* = 1.000).

Effects of disorder introduced by lithiation in the crystal structure of RMO can conveniently be observed through a two-stage evolution of the [310], [210], [020], and [111] peaks. At 10 K, these diffraction peaks broaden and separate in the first stage of lithiation from *x* = 0.000 to *x* = 0.375. Then these peaks sharpen at *x* = 0.500, and this effect is still pronounced at the higher temperature of 300 K. Upon further lithiation, this trend repeats itself in a second stage, from *x* = 0.625 to *x* = 0.875, when the diffraction peaks again start to broaden and then sharpen at *x* = 1.000. Nevertheless, the overall changes in the XRD patterns at the two different temperatures are less pronounced compared to what has been found from the radial distribution functions, see Figure 4. This is because the Mn species in the host material are much heavier (i.e., more electron rich) than the intercalating Li^+^ ions, providing much stronger X-ray scattering effect compared to the Li^+^ ions.

### 3.5 Voltage and Entropy Profiles

#### 3.5.1 Characterization of Voltage Profile

**Figure 7:**
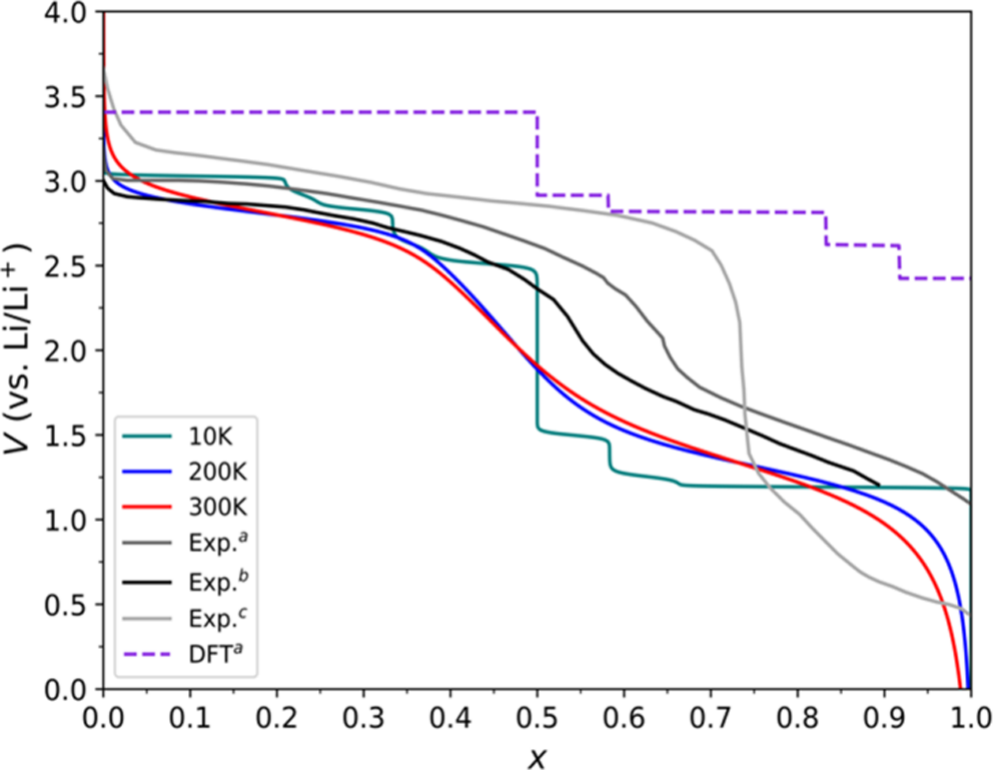
GCE derived (this work for different temperatures), experimentally observed ^a^ref (25), ^b^ref (29), ^c^ref (13) and DFT calculated ^a^ref (25) discharging voltage profiles as a function of lithium concentration, *x* in Li_*x*_MnO_2_.

Calculated voltage profiles are shown in Figure 7 along with available experimental^13,25,29^ and computational data^25^ obtained using periodic DFT calculations as mentioned in the Introduction. All profiles are monotonically decreasing with a gradient (slope) that is dependent upon the method used, temperature and the concentration of lithium. At 10 K, our calculated voltage profile consists of a series of plateaus connected by steps rather than smooth slopes, which may be a consequence of using a relatively small simulation cell. A small simulation cell constrains the possible configurations that can be sampled, therefore, restricting the investigation to a limited number of thermodynamically accessible structures in the ensemble obtained from our Monte Carlo supercell calculations, with only a few configurations near the global minima contributing to the final voltage profile. A similar behavior is also observed in the DFT profile, which only considered the global minima contributions to the voltage, i.e., employed an athermal model. In this study, at 10 K, we observe notable voltage drops at ca. *x* = 0.21, 0.33, 0.50, and 0.58, corresponding to RMOs with 5, 8, 12, and 14 lithium ions, respectively. However, when higher-energy structures become thermally accessible as temperature increases, this stepwise landscape is transformed to the blue (200 K) and red (300 K) curves shown in Figure 7. We note that the observed small voltage drops may also vary with the size of the chosen simulation cell; in particular, at low temperatures, the drop in voltage occurs at certain integers, matching with the number of lithium ions in the cell. Although, these small voltage drops may not have a significant impact on the voltage profile at working temperatures, it could be of fundamental interest to repeat these calculations in future on a larger simulation cell.

The most notable characteristic of a sharp voltage drop (from 2.5 to 1.2 V) is found in the voltage profiles within the concentration range of 0.4 < *x* < 0.6 and across all temperatures considered in our work, which is also predicted in the DFT investigation.^25^ This sharp drop is closely linked to the preference for lithium ion ordering in RMO at *x* = 0.5, as discussed in our RDF and XRD analyses above, of which the energy penalty of inserting lithium ions above this particular concentration becomes significant as it involves the shift of lithium ions from stable tetrahedral sites to unstable (in the limit of infinite dilution) octahedral sites, leading to significant changes in the ordering of lithium ions. Another sharp drop (but less significant) can be seen at *x* = 0.21 and 0.33 (10 K); however, this drop dwindles away rapidly as temperature increases, indicating that the energy penalty of adding lithium ions at this concentration is less than when *x* = 0.500, which is evident since the 2 × 1 channels have more space to accommodate lithium ions. An extreme example of such a drop is observed in λ-MnO_2_ (spinel) when *x* exceeds 0.5 in Li_*x*_MnO_2_, incurring a significant energy cost as lithium ions shift from tetrahedral sites to octahedral.^72,73^ Furthermore, similar drops have also been reported in α-MnO_2_, NaCoO_2_, and nickel–manganese-cobalt cathodes.^74−77^

Compared to the voltage profiles obtained here that are based on simulations, the voltage can be seen to drop at higher values of *x* in experiments, i.e., further to the right of *x* = 0.5. We note that formation of other lithiated MnO_2_ polymorph domains in partially lithiated RMO has been seen experimentally, which led us to speculate that lithiated spinel (λ) or intergrown (γ) domains would coexist in the cathode material and would, therefore, necessarily influence the voltage profile.^13,71,78,79^ For instance, as lithiation progresses, the coexisting phase domains will compete to accommodate lithium ions, intercalating into or out of interstitial (tetrahedral/octahedral) spinel sites. An extreme case of such a voltage drop, which occurs at ca. *x* = 0.9, has also been reported for γ-MnO_2_, where β and R phases coexist. In this case, the 1 × 1 channels in β and the 2 × 1 channels in R phases compete for lithium ions.^73,80^

#### 3.5.2 Order–Disorder Phase Transformations

**Figure 8:**
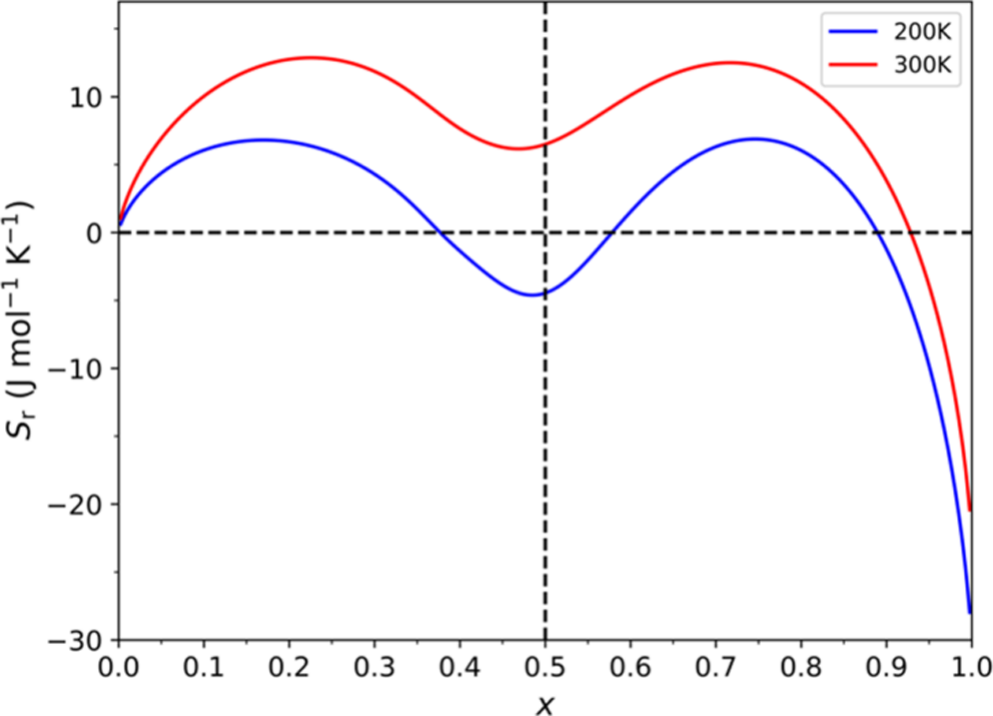
GCE derived entropy as a function of lithium concentration *x* in Li_*x*_MnO_2_ at different temperatures.

Finally, we investigate the change in entropy upon lithiation, which we report in Figure 8. We observe binodal entropy profiles, which are also commonly observed in intercalation-type electrodes. Such behavior is usually explained by the intercalation reaction involving two-step phase transformations.^81−83^ In this study, we identify three distinctive ordered or semiordered phases at lithium concentrations of *x* = 0.000, 0.500, and 1.000.

Considering the entropy profiles in Figure 8, as the concentration of lithium ions increases from *x* = 0.000, we first observe an increase in entropy. This initial increase in entropy is facilitated by the introduction of defects in a pure RMO resulting in a disordered structure. Subsequently, the increase in entropy reaches its maximum at the first node, occurring at ca. *x* = 0.17 at 200 K and ca. *x* = 0.23 at 300 K. A higher entropy is achieved for the higher temperature as more states are accessible both because of the higher temperature and because of the higher concentration of lithium that leads to a greater disordered structure. Once the first maximum is reached, the entropy starts to drop with increasing concentration and stabilizes at around *x* = 0.500, which marks the first phase transformation. At this concentration, we observe the ordered structure where the lithium ions have intercalated in stable tetrahedral sites, as depicted in Figure 4d, or Figure 5a with a {3,3,3,3} arrangement.

In the second range, 0.500 < *x* < 1.000, as more lithium ions are added, we again observe that entropy initially increases. This increase in entropy is related to the disorder in the arrangement of lithium ions caused by the newly added lithium ions shifting the ions already occupying the tetrahedral sites to unstable octahedral sites. Again, there is a maximum in the entropy before it falls to a minimum at *x* = 1.000, marking the second phase transformation. At this maximum concentration, the structure corresponds to a fully lithiated RMO with lithium ions at octahedral sites, as depicted in Figure 4f, or Figure 5b with a {6,6,6,6} arrangement.

## 4 Conclusions

In this investigation, we provide a proof-of-concept study on lithiated R-MnO_2_ (ramsdellite), demonstrating how an unbiased Monte Carlo technique combined with an interatomic potential method can be efficiently used to simulate solid solutions using a grand canonical ensemble approach. Previously we have applied this method to investigate the stability of polar surfaces of ZnO^63^ and now have extended and developed the method and its implementation further to explore the electrochemical processes in a Li-battery cathode material, R-MnO_2_.

We have successfully modeled the charging/discharging voltage profile and X-ray diffraction patterns of partially lithiated R-MnO_2_, which agree with experiment and density functional theory results.^13,25,29^ Furthermore, we report a two-step phase transformation, marking the distinctive ordered or semiordered phases at *x* = 0, 0.5, and 1 in Li_*x*_MnO_2_, whereas the disordered phases are observed in the concentration ranges between these, i.e., 0 < *x* < 0.5 and 0.5 < *x* < 1.0, evidenced by the broadening/splitting in X-ray diffraction peaks, radial distribution functions and the calculated configurational entropy profiles. In particular, the ordered phase found at *x* = 0.5 is a critical point where the addition of more lithium ions (or higher temperature) drives preoccupying lithium ions from energetically stable tetrahedral sites onto the more numerous and previously unstable octahedral sites. The resulting rapid voltage drop is due to the energy penalty of adding lithium ions above this lithium concentration. The radial distribution functions show an ordering behavior of lithium ions in the 2 × 1 channels of R-MnO_2_, which minimizes the electrostatic energy of the system.

Possible extensions of the current study could include both methodological improvements and work on more complex cathode material systems, including intergrown structures of different phases as well as pure phases with low-dimensional defects such as stacking faults (De Wolff disorder).^84^ Furthermore, Jahn–Teller effects can be considered explicitly where, for example, the angular overlap model could be employed, an additional energy term to our total potential.^85−87^ Moreover, incorporation of the vibrational dynamic effects should be investigated and presents the next challenge.

Our findings provide crucial insights into the behavior of R-MnO_2_ as a cathode material, particularly the importance of lithium ion ordering, voltage characteristics, and structural transformations. The outcomes from this study will be beneficial for designing future high-capacity Mn based lithium ion batteries. The developed methodology is based on a systematic exploration of the configurational space spanned by a solid solution exemplified here with partially lithiated R-MnO_2_ that enables a calculation of accurate thermodynamic potentials of the relevant grand canonical ensemble. This developed and tested method has advantages over traditional statistical approximation approaches like that of cluster expansion and inversion methods; in particular, our approach can generate more accurate energy distributions because our method accounts for important long-range Coulomb interactions and, therefore, fluctuations. Any measure that results from the predicted distributions, e.g., the X-ray diffraction pattern, will be more reliable.

## Supporting Information Available

Calculated defect structures of lithium doped ramsdellite MnO_2_ in the infinite dilution limit, and details of the Mott-Littleton energy convergence as a function of cutoff radius (PDF)
