# Supplementary material for: Discharging of Ramsdellite MnO2 Cathode in a Lithium-Ion Battery
Source: Chem Mater. 2024 Aug 30;36(18):8737–52. doi: 10.1021/acs.chemmater.4c01417 (PMC11428077; doi:10.1021/acs.chemmater.4c01417)
Supplement: Supplementary file 1 — cm4c01417_si_001.pdf [file cm4c01417_si_001.pdf]

Supporting Information

Discharging of Ramsdellite MnO_2 Cathode in a Lithium-Ion Battery

Woongkyu Jee,[a] Alexey A. Sokol,[a] Cyril Xu,[a] Bruno Camino,[a] Xingfan Zhang,[a] Scott M. Woodley*[a]

[a] Department of Chemistry, University College London, 20 Gordon Street, London WC1H 0AJ, United Kingdom

* Correspondence author: scott.woodley@ucl.ac.uk

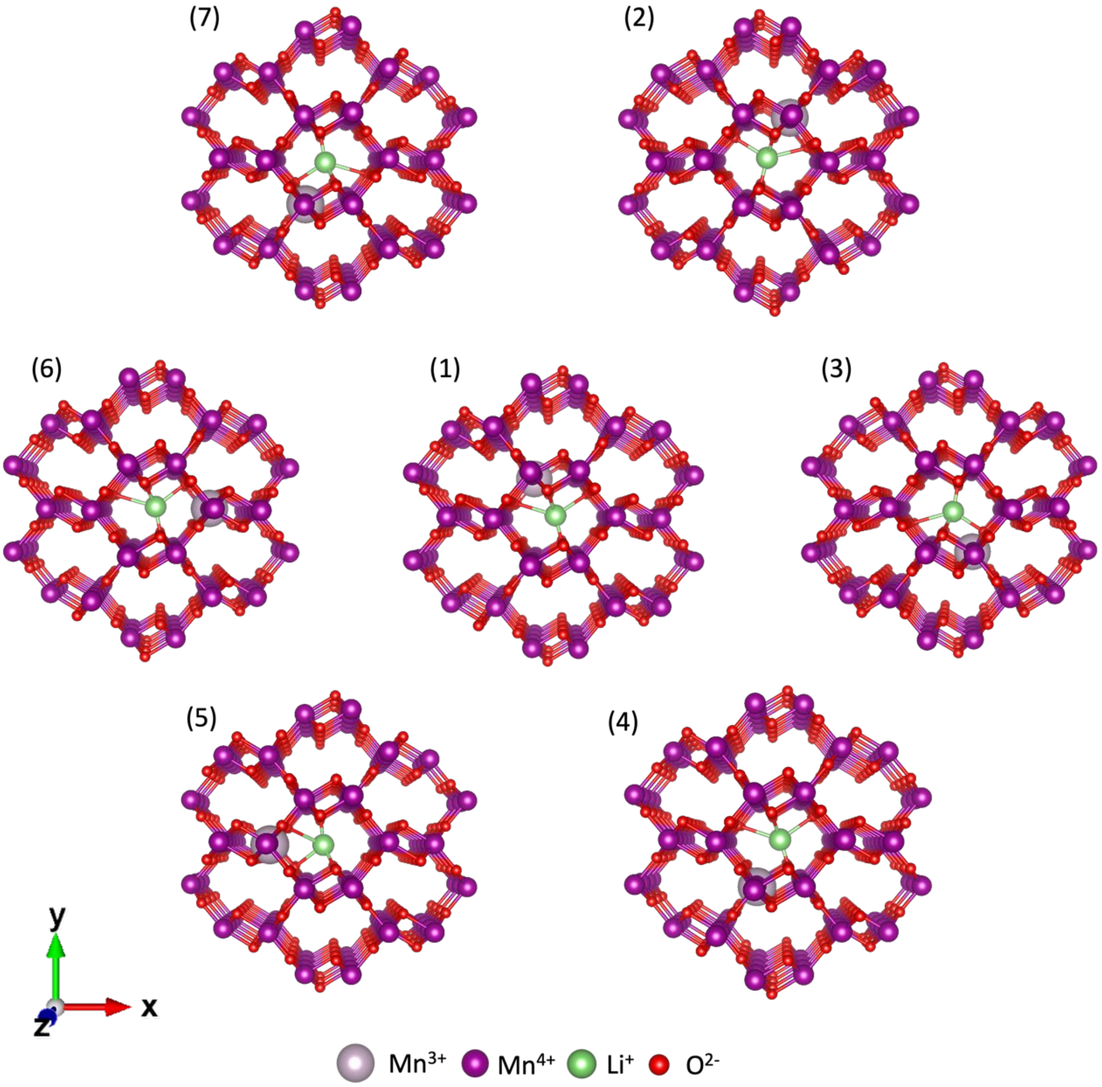

Figure S1. Local defect structures in lithium doped ramsdellite MnO_2. The structures were obtained from the Mott-Littleton calculations as described in Section 2.3 of the main text. Ball-and-stick model is used to represent respective ions: Mn^{3+} (grey), Mn^{4+} (purple), Li^+ (green) and O^{2-} (red).

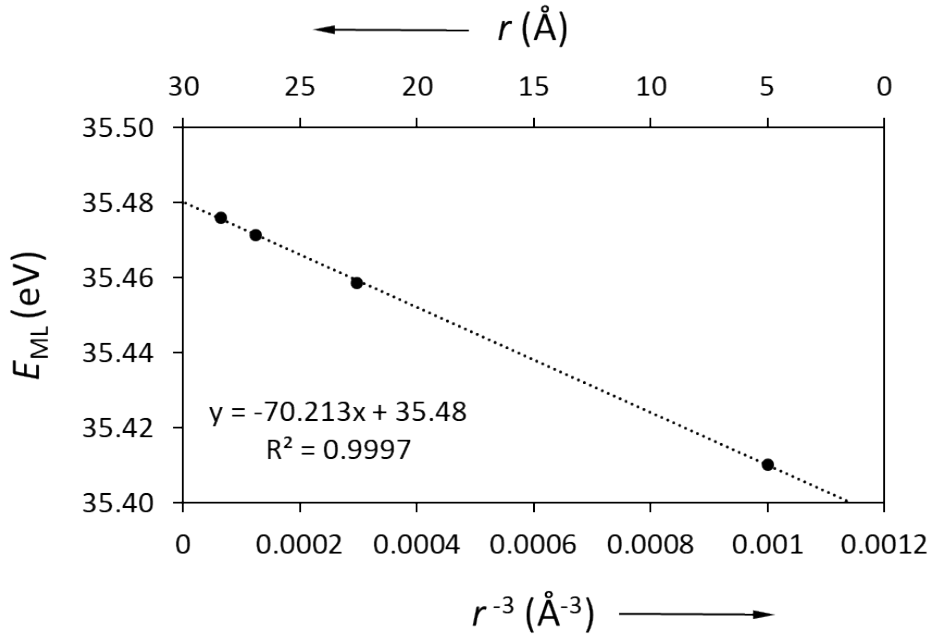

Figure S2. Mott-Littleton energy convergence plot, showing the calculated defect energies as a function of region I size, whilst constraining the thickness of region IIa to 25 Å. The trendline indicates a linear dependence on $1/r^3$, which allows extrapolation as r tends to infinity. Data were obtained using structure (1) in Fig S1.
